# Effects of Ganodermanondiol, a New Melanogenesis Inhibitor from the Medicinal Mushroom *Ganoderma lucidum*

**DOI:** 10.3390/ijms17111798

**Published:** 2016-10-27

**Authors:** Ji-Woong Kim, Hong-Il Kim, Jong-Hyeon Kim, O-Chul Kwon, Eun-Suk Son, Chang-Soo Lee, Young-Jin Park

**Affiliations:** Department of Biomedical Chemistry, Konkuk University, Chungju 27478, Korea; jumpbook@naver.com (J.-W.K.); kwangdae7@kku.ac.kr (H.-I.K.); dhqksdl@kku.ac.kr (J.-H.K.); ntty212@daum.net (O.-C.K.); eunsuk0607@kku.ac.kr (E.-S.S.); cslee@kku.ac.kr (C.-S.L.)

**Keywords:** B16F10 melanoma cell, *Ganoderma lucidum*, ganodermanondiol, microphthalmia-associated transcription factor, tyrosinase, tyrosinase-related proteins

## Abstract

*Ganoderma lucidum*, a species of the Basidiomycetes class, has been attracting international attention owing to its wide variety of biological activities and great potential as an ingredient in skin care cosmetics including “skin-whitening” products. However, there is little information available on its inhibitory effect against tyrosinase activity. Therefore, the objectives of this study were to investigate the chemical composition of *G. lucidum* and its inhibitory effects on melanogenesis. We isolated the active compound from *G. lucidum* using ethanol extraction and ethyl acetate fractionation. In addition, we assayed its inhibitory effects on tyrosinase activity and melanin biosynthesis in B16F10 melanoma cells. In this study, we identified a bioactive compound, ganodermanondiol, which inhibits the activity and expression of cellular tyrosinase and the expression of tyrosinase-related protein-1 (TRP-1), TRP-2, and microphthalmia-associated transcription factor (MITF), thereby decreasing melanin production. Furthermore, ganodermanondiol also affected the mitogen-activated protein kinase (MAPK) cascade and cyclic adenosine monophosphate (cAMP)-dependent signaling pathway, which are involved in the melanogenesis of B16F10 melanoma cells. The finding that ganodermanondiol from *G. lucidum* exerts an inhibitory effect on tyrosinase will contribute to the use of this mushroom in the preparation of skin care products in the future.

## 1. Introduction

Natural products have been used for centuries to promote healthy skin. Currently, they are becoming more prevalent in commercial formulations owing to consumers’ concerns about synthetic substances and a greater market demand for natural ingredients. Despite the widespread use of natural ingredients, the discovery of biologically active compounds, the determination of the specific identity of the chemicals with desirable effects, and the development of these substances into new cosmetic products remain an important challenge.

Melanin, a black pigment synthesized from tyrosine by epidermal melanocytes, is the main determinant of hair and skin color [1,2,3,4,5,6]. Tyrosinase (EC 1.14.18.1), a multifunctional copper-containing oxidase, is considered the key enzyme that orchestrates melanogenesis in melanocytes. This key enzyme catalyzes both the hydroxylation of l-tyrosine and the oxidation of l-3,4-dihydroxyphenylalanine (l-DOPA) to *o*-quinone (dopaquinone) [7]. To treat epidermal hyperpigmentation conditions, a multitude of different tyrosinase inhibitors have been used to date. In the cosmetics industry, bioactive compounds specifically available for skin whitening include hydroquinone (HQ) [8], retinol [9], azelaic acid [10], kojic acid [11], vitamin C [12], arbutin [13], and other botanicals. HQ, the most popular depigmenting agent, inhibits the conversion of DOPA to melanin by inhibiting the activity of tyrosinase [14]. However, the cytotoxic and mutagenic effects of HQ on melanocytes as well as on mammalian cells have been demonstrated in previous studies [15,16]. Therefore, the use of HQ in cosmetics has been banned in numerous countries owing to health concerns. Although this has led to the development of alternative agents such as kojic acid and arbutin, their potential use in cosmetic products appears to be limited by their adverse effects and low stability [17].

Various plants have been the main source of all cosmetics before the availability of synthesized substances with similar properties [18]. Several medicinal plants (traditional herbal medicines) were found to have dual uses as both curative and cosmetic agents. Indeed, some traditional herbal medicines have been investigated for their potential use in various cosmetic products [19,20]. In addition to plants, macrofungi (mushrooms) also contain notable biomedicinal agents, which have numerous health benefits [21].

*Ganoderma lucidum* (Curt.: Fr.) P. Karst. (Lingzhi in Chinese, Reishi in Japanese, and Youngzi in Korean), a species of the Agaricomycetes class and the family Ganodermataceae, is one of the most popular medicinal mushrooms in Korea, China, and Japan, as well as other regions of the world [22]. It has attracted international attention as a valuable medicinal agent because of its wide variety of antitumor, antiperoxidative, anti-inflammatory, antimutagenic, antioxidant, and other biological activities [23,24]. *G. lucidum* has applications as a food additive and active pharmaceutical agent, and great potential as a cosmetic ingredient [25]. The extracts of this mushroom have been used as a raw material for their beneficial properties in the manufacture of cosmetic products, an interesting, innovative approach for the cosmetics industry.

The name *Ganoderma* is derived from the Greek ganos (meaning “brightness” and “sheen”) and derma (meaning “skin”). Unsurprisingly, several whitening facial mask products on the market currently contain *Ganoderma* extracts as their ingredients. The rationale for this is the fact that *G. lucidum* extract has low cytotoxicity and a strong whitening effect. Chien et al. [25] reported the inhibition of tyrosinase activity of the extracts of several mushrooms including *G. lucidum*, *Antrodia camphorat*, *Agaricus brasiliensis*, and *Cordyceps militaris*. Extracts of *G. lucidum* exhibit inhibitory activities on tyrosinase and do not show any toxicity against human fibroblasts Hs68 [25]. However, there are currently no reports on the inhibition of tyrosinase by bioactive compounds in *G. lucidum*, which could be direct mediators of the “skin-whitening” effects. Therefore, the purpose of the present study was to isolate a tyrosinase inhibitor from *G. lucidum* and evaluate its biological function in a B16F10 melanoma model in an effort to explain its skin-whitening effects.

Here, we first report the results of our investigation of the whitening effect of ganodermanondiol as a tyrosinase inhibitor present in the medicinal mushroom *G. lucidum*. Ganodermanondiol significantly inhibited tyrosinase activity, the expression of tyrosinase-related proteins, and microphthalmia-associated transcription factor (MITF) expression in B16F10 melanoma cells. These results suggest that ganodermanondiol from *G. lucidum* may be a potential candidate for development as an anti-pigmenting agent.

## 2. Results and Discussion

### 2.1. Chemical Structure and Cytotoxicity of Ganodermanondiol Isolated from Ganoderma lucidum on B16F10 Melanoma Cells

**Figure 1:**
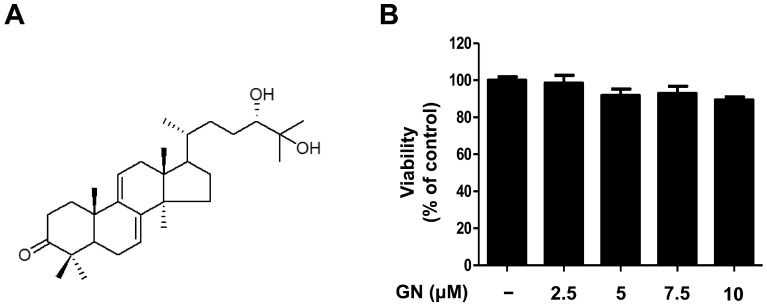
Chemical structure and cytotoxicity of ganodermanondiol (GN) isolated from *G. lucidum.* (**A**) Chemical structure; (**B**) cytotoxic effects of GN on B16F10 melanoma cells. B16F10 cells were treated with various concentrations of GN (2.5, 5, 7.5, and 10 μM) for 24 h. Values are means ± standard deviation (SD) of three independent experiments and relative to percentages of control cells.

Previous studies of *G. lucidum* have established that it contains more than 300 biologically active compounds such as triterpenoids, polysaccharides, and steroids [26,27]. Initially, the ethanol (EtOH) extract of dried *G. lucidum* was suspended in water and partitioned successively with ethyl acetate (EtOAc). Using bioassay-guided fractionation, the EtOAc-soluble fraction was subjected to repeated column chromatography to afford ganodermanondiol. The ganodermanondiol was identified by comparing its spectroscopic nuclear magnetic resonance (NMR) data (Figure 1A) with those reported in the literature [28], and the results showed they were identical. Ganodermanondiol has a triterpenoid structure and is one of the major active components of *G. lucidum* [28]. It has a variety of biological effects including inhibitory activity against human immunodeficiency virus (HIV)-1 protease, anti-complement activity, and hepatoprotective action [26,27,28]. However, other biological activities, especially its skin-related activity, of ganodermanondiol remain to be elucidated. Therefore, in this study, we determined the cytotoxic effects of ganodermanondiol on B16F10 melanoma cells treated with indicated concentrations for 24 h. Concentrations of 2.5, 5, 7.5, and 10 μM ganodermanondiol showed no cytotoxic effects in the 3-(4,5-dimethylthiazol-2-yl)-2,5-diphenyltetrazolium bromide (MTT) assay (Figure 1B). Therefore, the B16F10 cells were treated with ganodermanondiol at concentrations of 2.5, 5, 7.5, and 10 μM in subsequent experiments.

### 2.2. Effects of Ganodermanondiol on Melanin Contents and Tyrosinase Activity of B16F10 Cells

**Figure 2:**
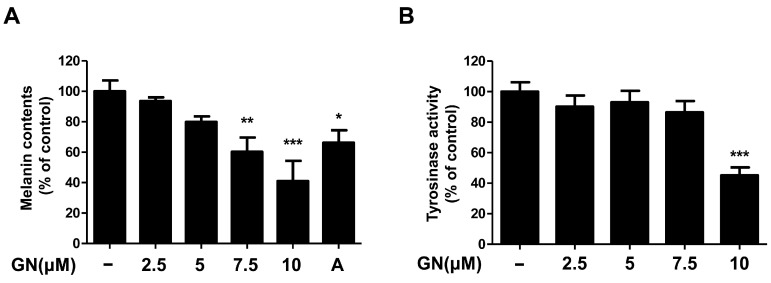
Effects of ganodermanondiol (GN) on melanin production and cellular tyrosine activity of B16F10 cells. GN inhibits (**A**) melanin production and (**B**) cellular tyrosinase activity in B16F10 cells treated with various concentrations (2.5, 5, 7.5, and 10 μM) for 72 h. Values are means ± standard deviation (SD) of three independent experiments. Statistical significance of differences was evaluated using a one-way analysis of variance (ANOVA) followed by Tukey’s test. * *p* < 0.05, ** *p* < 0.01, and *** *p* < 0.001 versus B16F10 cells without GN treatment. A: arbutin (0.5 mM).

Melanin plays a pivotal role in protecting human skin, especially keratinocytes against environmental damage including ultraviolet (UV) radiation, heat, and solar radiation [1,29]. Melanin is the main determinant of skin color variation, which is due to the melanin content of the skin of the human body. Melanin content levels correlate directly with the activity of tyrosinase [30], which is an enzyme that regulates melanin synthesis. Tyrosinase inhibitors are used as human skin-whitening agents based on their inhibition of melanogenesis [12]. Therefore, in this study, we demonstrated the mechanisms of melanogenesis inhibition by ganodermanondiol isolated from *G. lucidum* in B16F10 cells treated with various concentrations (2.5, 5, 7.5, and 10 μM) for 72 h. Treatment with ganodermanondiol significantly reduced the melanin content of B16F10 cells in a dose-dependent manner (Figure 2A). In addition, we also examined the effects of ganodermanondiol on tyrosinase activity, which was shown to decrease in B16F10 cells after treatment with ganodermanondiol (Figure 2B). These findings indicate that ganodermanondiol significantly inhibited the production of melanin and tyrosinase activity.

### 2.3. Effects of Ganodermanondiol on Cellular Melanogenesis-Related Proteins and MITF Protein Expression in B16F10 Cells

**Figure 3:**
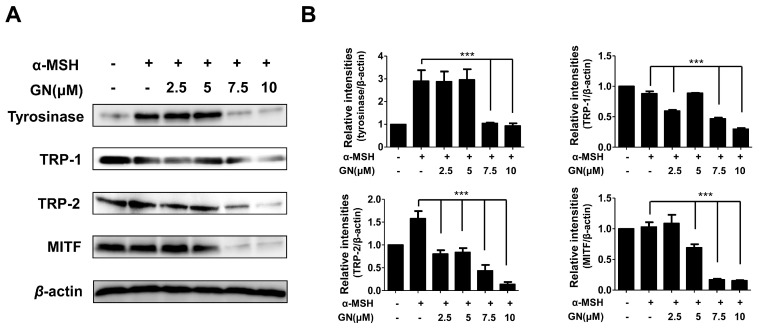
Effects of ganodermanondiol (GN) on cellular melanogenesis-related protein and microphthalmia-associated transcription factor (MITF) protein expression in B16F10 cells, pretreated with various concentrations of GN (2.5, 5, 7.5 and 10 μM) for 24 h following treatment with or without α-melanocyte-stimulating hormone (α-MSH) for 24 h. (**A**) Cellular proteins levels were examined using Western blot analysis; (**B**) Relative protein expression levels of tyrosinase, tyrosinase related protein 1 (TRP-1), TRP-2, and MITF protein. Relative protein expression levels were calculated using TL-100 software program (TotalLab, Newcastle, UK). Values are means ± standard deviation (SD) of three independent experiments. Statistical significance of differences was evaluated using a one-way analysis of variance (ANOVA) followed by Tukey’s test. *** *p* < 0.001 versus B16F10 cells without GN treatment.

Tyrosinase-related enzymes including tyrosinase, tyrosinase-related protein-1 (TRP-1), and TRP-2 are key factors in the biosynthesis of melanin [1]. These enzymes are important for regulating melanogenesis pathways, and they constitute a specific family of membrane proteins [1]. Importantly, tyrosinase protein functions as a catalyst for the rate-limiting reaction in the melanogenesis pathways. TRP-1 and TRP-2 are believed to determine the shape of melanosomes, which are the structures in melanocytes where melanin is produced [31]. In our study, to elucidate the mechanisms underlying the inhibition of melanin production and tyrosinase activity by ganodermanondiol, we examined the effects of ganodermanondiol on cellular tyrosinase protein, as well as TRP-1 and TRP-2 expression, which are all associated with melanogenesis. Western blot analysis showed that ganodermanondiol inhibited the expression of tyrosinase protein, TRP-1, and TRP-2 in B16F10 cells. The induction of these proteins by α-melanocyte-stimulating hormone (α-MSH) was markedly decreased by pre-treatment with ganodermanondiol (Figure 3). These findings indicate that the inhibitory action of ganodermanondiol on melanogenesis is associated with the suppression of the expression of cellular melanogenesis-related proteins including tyrosinase protein, TRP-1, and TRP-2.

**Figure 4:**
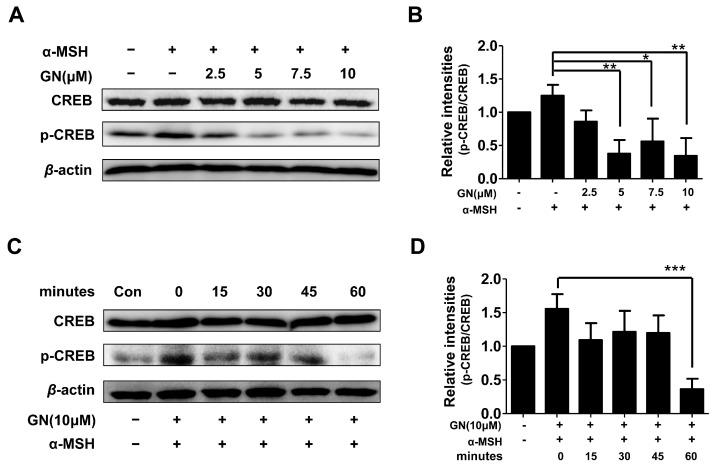
Effects of ganodermanondiol (GN) on phosphorylation of cyclic adenosine monophosphate (cAMP) response element binding protein (CREB) in B16F10 cells, pretreated with various concentrations of GN (2.5, 5, 7, and 10 μM) for 24 h following treatment with or without α-melanocyte-stimulating hormone (α-MSH) for 24 h. Cellular protein levels were examined using Western blot analysis with (**A**) various concentrations of GN (2.5, 5, 7 and 10 μM) and (**C**) at different times (0, 15, 30, 45, and 60 min); (**B**) Relative phosphorylation levels of CREB protein with various concentrations of GN and at (**D**) different times were calculated using TL-100 software program (TotalLab, Newcastle, UK). Values are presented as mean ± standard deviation (SD) of three independent experiments. Statistical significance of differences was evaluated using a one-way analysis of variance (ANOVA) followed by Tukey’s test. * *p* < 0.05, ** *p* < 0.01 and *** *p* < 0.001.

Various cellular transcription factors are involved in melanogenesis, and the most notable is MITF [32]. Generally, melanogenesis directly leads to increased MITF expression through the activation of tyrosinase enzyme, TRP-1, and TRP-2, as well as tyrosinase activity, resulting in increased melanin synthesis. Moreover, the downregulation of MITF expression through tyrosinase, TRP-1, and TRP-2 is the most significant mechanism underlying the anti-melanogenic effects of inhibitory agents [32]. Therefore, we investigated whether ganodermanondiol-regulated melanogenesis occurs via an MITF expression pathway. To assess the correlation of MITF regulation with a reduction in cellular melanogenesis-related protein expression by ganodermanondiol treatment, we investigated MITF protein expressions in B16F10 cells using an anti-MITF antibody. MITF protein expression was decreased by pretreatment with ganodermanondiol in α-MSH-stimulated B16F10 cells (Figure 4). Therefore, we suggest that the reduction in melanogenesis-related protein expression including tyrosinase protein, TRP-1, and TRP-2 by ganodermanondiol treatment is mediated by the MITF pathway in B16F10 cells.

### 2.4. Effects of Ganodermanondiol on cAMP Response Element Binding Protein (CREB) Phosphorylation in B16F10 Cells

As described above, ganodermanondiol significantly inhibited tyrosinase, TRP-1, and TRP-2 protein expression by downregulating MITF. Tyrosinase, TRP-1, and TRP-2 expression is primarily regulated by MITF [32]. It has been reported that MITF downregulation is related to the suppression of the cAMP-dependent melanogenic pathway stimulated by a-MSH in melanoma cells. The α-MSH binds to and stimulates the melanocortin-1 receptor (MC1R) [33], which upregulates intracellular cAMP levels and induces melanogenesis through cellular signaling in melanocytes [34]. cAMP activates protein kinase A (PKA), which subsequently phosphorylates CREB protein, thereby upregulating MITF expression. The *MITF* gene itself is a target of cAMP signaling induced by α-MSH, and predominantly regulates tyrosinase and TRP-1 protein expression [35,36,37]. In the cAMP-dependent signaling pathway, the dual phosphorylation of the cAMP response element binding protein (CREB), mediated by the kinase activities of ribosomal S6 kinase (RSK) and PKA, has been found to activate MITF transcription [38,39]. Furthermore, it has been reported that suppression of the cAMP-dependent signaling pathway causes MITF degradation in α-MSH-stimulated melanoma cells [40]. Therefore, we hypothesized that GN-induced MITF downregulation is involved in the cAMP signal transduction cascade. As expected, the phosphorylation of CREB proteins was significantly decreased by pretreatment with ganodermanondiol in the α-MSH-stimulated B16F10 cells in a dose- and time-dependent manner (Figure 4). Although, PKA activation along with cAMP levels were not determined in the present study, these results suggest that the inhibitory effect of ganodermanondiol contributed to the reduction in MITF expression and melanin production through the inhibition of CREB phosphorylation.

### 2.5. Effects of Ganodermanondiol on Phosphorylated (p)-p38, p-c-Jun N-Terminal Kinase (JNK) and p-Extracellular Signal-Regulated Kinase (ERK) Protein Levels in B16F10 Cells

Melanogenic activation by binding stem cell factor (SCF) and endothelin-1 (EDN1) to c-kit and endothelin receptor type B (EDNRB) receptor, respectively, have been well studied in melanocytes [41]. Binding END1 to EDNRB activates protein kinase C (PKC) by released diacylglycerol (DAG) from the cell membrane. The activated PKC then phosphorylates RAF proto-oncogene serine/threonine-protein kinase (Raf-1). On the other hand, SCF triggers dimerization and autophosphorylation of c-kit receptors followed by activation of various substrates and conversion of rat sarcoma-guanosine diphosphates (Ras-GDP) to RAS-guanosine triphosphate (GTP), leading to phosphorylation of Raf-1. The phosphorylation of Raf-1 activates (phosphorylates) a series of mitogen-activated protein kinases (MEKs), extracellular signal-regulated kinases (ERKs), RSKs, and CREB in the mitogen-activated protein kinase (MAPK) cascade [41]. In addition, the MAPK family proteins including ERK, p38, and c-Jun N-terminal kinase (JNK) are also known to play crucial roles in melanogenesis [39]. The phosphorylation of p38 induces MITF expression, whereas the phosphorylation of ERK and JNK downregulate melanin synthesis [39].

**Figure 5:**
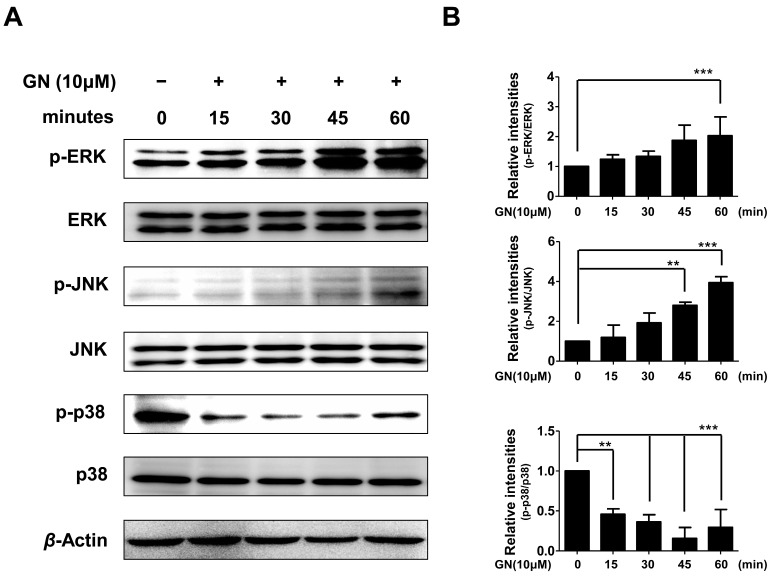
Effects of ganodermanondiol (GN) on phosphorylation of extracellular signal-regulated kinases (ERKs), c-Jun N-terminal kinases (JNKs), and p38 proteins in B16F10 cells, treated with GN (10 μM) for 0, 15, 30, 45, and 60 min. Cellular protein levels were examined using (**A**) Western blot analysis; and (**B**) relative phosphorylation levels were calculated using the TL-100 software program (TotalLab, Newcastle, UK). Values are means ± standard deviation (SD) of three independent experiments. Statistical significance of differences was evaluated using a one-way analysis of variance (ANOVA) followed by Tukey’s test. ** *p* < 0.01 and *** *p* < 0.001.

Therefore, in the present study, we also evaluated whether the effects of ganodermanondiol on phosphorylation of these proteins involved the MAPK family proteins. As shown in Figure 5, the phosphorylation of ERK and JNK proteins were significantly increased after 60 and 45 min, respectively, by treatment with ganodermanondiol (10 µM), whereas 15 min after treatment, phosphorylation of p38 protein was significantly decreased in B16F10 cells. These results indicate that the inhibition of melanogenesis by ganodermanondiol is also associated with MAPK family proteins. In particular, ganodermanondiol induced the phosphorylation of ERK and JNK; however, phosphorylation of p38 was suppressed by ganodermanondiol treatment.

## 3. Materials and Methods

### 3.1. Reagents

Dulbecco’s modified Eagle’s medium (DMEM), fetal bovine serum (FBS), antibiotics, and other tissue culture reagents were acquired from Gibco BRL (Grand Island, NY, USA). Primary antibodies against TRP-1, TRP-2, tyrosinase, and actin were obtained from Santa Cruz Biotechnology (Dallas, TX, USA). 3-(4,5-Dimethylthiazol-2-yl)-2,5-diphenyltetrazolium bromide (MTT), α-MSH, and all other chemicals were purchased from Sigma-Aldrich (St. Louis, MO, USA).

### 3.2. G. lucidum

*G. lucidum* was purchased from Humanfood, Imsil-eup, Iimsil-gun, Jeonbuk Province, Korea, in 2014. A voucher specimen (No. KU12-008) has been deposited in the Department of Biomedical Chemistry, Konkuk University, Chungju, Korea.

#### Isolation of Ganodermanondiol

Dried *G. lucidum* (4 kg) was crushed and extracted with EtOH (30 L) at room temperature for 7 days. After evaporating the solvent in vacuo, the extract (90 g) was treated with water (2 L) and extracted thrice with EtOAc (each time 3 L) to obtain the EtOAc-soluble fraction (70 g). The EtOAc-soluble fraction (70 g) was separated on a silica gel (230–400 mesh, Merck Millipore, Darmstadt, Germany) column using *n*-hexane:EtOAc (gradient, 20:1–1:1) and chloroform:methanol (MeOH) (gradient, 20:1–1:1) to obtain 10 fractions (Fr. 1–10). Using bioassay-guided fractionation, Fractions 5 and 6 were combined and separated using a silica gel (70–230-mesh, Merck Millipore, Darmstadt, Germany) column using a chloroform:EtOAc (10:1–5:1) to obtain four fractions (Fr. A–D). Fraction D was subsequently purified using a reverse phase (RP) C-18 (Merck Millipore, Darmstadt, Germany) column chromatography with acetonitrile:water (1:1–7:3) to give ganodermanondiol (10.5 mg, purity >98.0%).

The ganodermanondiol was identified by comparing the spectroscopic NMR data of the isolated sample with that previously reported [28] and both were identical. For each experiment, ganodermanondiol was dissolved in dimethylsulfoxide (DMSO, final culture concentration 0.05%).

Ganodermanondiol showed the following characteristics: Colorless, C_30_H_48_O_3_.

Proton (^1^H)-NMR (CDCl_3_, 300 MHz): δ 0.59 (3H, s, H-18), 0.87 (3H, s, H-28), 0.91 (3H, d, *J* = 6.3 Hz, H-21), 1.08 (3H, s, H-19),1.12 (3H, s, H-29), 1.16 (3H, s, H-26), 1.19 (3H, s, H-27), 2.77 (1H, m, H-1), 3.30 (1H, bd, *J* = 8.1 Hz, H-24), 5.38 (1H, bd, *J* = 6.3 Hz, H-7), 5.50 (1H, bd, *J* = 6.3 Hz, H-11). ^13^C-NMR (75 MHz, CDCl_3_): δ 15.70 (C-18), 18.61 (C-21), 22.02 (C-19), 22.43 (C-30), 23.19 (C-28), 23.64 (C-6), 25.32 (C-27), 25.42 (C-26), 26.54 (C-29), 27.83 (C-16), 28.69 (C-15), 31.44 (C-22), 33.45 (C-23), 34.83 (C-2), 36.51 (C-20), 36.51 (C-20), 36.60 (C-1), 37.17 (C-12), 37.80 (C-10), 43.73 (C-13), 47.45 (C-4), 50.28 (C-5), 50.69 (C-14), 50.95 (C-17), 73.22 (C-25), 79.57 (C-24), 117.23 (C-11), 119.90 (C-7), 142.83 (C-8), 144.49 (C-9), 216.83 (C-3) [24].

### 3.3. Cell Culture

B16F10 murine melanoma cells (CRL-6475, American Type Culture Collection (ATCC), Manassas, VA, USA) were cultured in DMEM supplemented with 10% FBS and 1% penicillin (all Thermo Fisher Scientific Inc., Waltham, MA, USA). The cells were incubated in a humidified atmosphere of 95% air and 5% CO_2_ at 37 °C.

### 3.4. Cell Viability Assay

The viability of B16F10 melanoma cells was evaluated using an MTT assay [42]. Cells were incubated with various concentrations of the ganodermanondiol (2.5, 5, 7.5, and 10 μM) for 24 h. For determination of cell viability, 50 mg/mL of MTT solution incubated with a cell suspension for 3 h, and the resulting formazan crystals were dissolved in DMSO. The optical density (OD) was measured at 570 nm using a microplate reader (TECAN, Männedorf, Switzerland).

### 3.5. Tyrosinase Activity in B16F10 Cells

Cellular tyrosinase activity was measured using a previously described method [43]. B16F10 cells were cultured in 100 mm dishes using DMEM and incubated at 37 °C in a humidified 5% CO_2_ incubator. After treatment with various concentrations of ganodermanondiol (2.5, 5, 7.5, and 10 μM) for 1 h, followed by treatment with 300 μM α-MSH for 72 h, the cells were harvested and lysed using phosphate-buffered saline (PBS, pH 6.8) containing 1% Triton X-100. The cells were disrupted by freezing and thawing, and the lysates were clarified by centrifugation at 10,000× *g* for 10 min. The protein content was determined using a bicinchoninic acid (BCA) protein assay kit (Pierce Biotechnology, Rockford, IL, USA). Each well of a 96-well flat-bottom plate contained 40 μg of protein, 2.0 mM l-DOPA, and 0.1 M PBS (pH 6.8). The plate was incubated at 37 °C for 1 h, and the absorbance was measured at 475 nm using a microplate reader. Tyrosinase activity was measured according to the following formula: tyrosinase activity (%) = (OD_475_ of sample/OD_475_ of control) × 100.

### 3.6. Melanin Contents of B16F10 Cells

Melanin content of the melanocytes was measured according to a previously described method [43]. B16F10 cells were cultured in six-well plates and treated with various concentrations of ganodermanondiol (2.5, 5, 7.5, and 10 μM) for 1 h, and then incubated for 72 h. After the medium was discarded, the wells were washed with PBS. Cells were incubated overnight in the dark in 1 M sodium hydroxide (NaOH) at 37 °C. The relative melanin content was measured using an enzyme-linked immunosorbent assay (ELISA) plate reader (TECAN, Männedorf, Switzerland) at 450 nm. The melanin content was expressed as picogram (pg) per cell. Arbutin (0.5 mM) was used as a standard under the same experimental conditions.

### 3.7. Western Blot Analysis

The cell pellets were lysed using radioimmunoprecipitation assay (RIPA) lysis buffer (50 mM Tris (pH 7.4), 150 mM sodium chloride (NaCl), 1 mM ethylenediaminetetraacetic acid (EDTA), and 1% NP40), and the protein concentration was determined using the Bradford assay reagent (Bio-Rad, Philadelphia, PA, USA). After the measurement, equal amounts of protein were separated using sodium dodecyl sulfate-polyacrylamide gel electrophoresis (SDS-PAGE) and transferred to a Hybond-enhanced chemiluminescence nitrocellulose membrane (Bio-Rad, Philadelphia, PA, USA). The membrane was blocked with 3% bovine serum albumin (BSA) and incubated with MITF, tyrosinase, TRP-1, TRP-2, CREB, p-CREB , ERK, p-ERK, p38, p-p38, JNK, and p-JNK antibodies (all 1:1000) at 4 °C overnight. The bands were visualized using horseradish peroxidase (HRP)-conjugated secondary antibodies (1:5000, Santa Cruz Biotech, Dallas, TX, USA) and enhanced chemiluminescence, and then quantified using the TL-100 software program. Representative blots of at least three independent experiments are presented.

### 3.8. Statistical Analysis

The data are expressed as mean ± standard deviation (SD) of at least three independent experiments. Significant differences relative to controls were evaluated using a one-way analysis of variance (ANOVA) followed by Tukey’s test using Prism (GraphPad Software Inc., La Jolla, CA, USA).

## 4. Conclusions

The results of the present study suggest that ganodermanondiol, a triterpenoid compound isolated from *G. lucidum*, effectively regulated melanogenesis in B16F10 melanoma cells. We found that ganodermanondiol inhibited the expression of cellular melanogenesis-related proteins including tyrosinase, TRP-1, and TRP2, as well as MITF in B16F10 cells. Moreover, ganodermanondiol also affected the MAPKs and the cAMP-dependent signaling pathway, which may contribute to the reduction in MITF expression, thereby leading to decreased melanin production. In recent years, many researchers have attempted to identify effective and safe anti-melanogenesis ingredients [44]. Melatonin [45,46], 4-hydroxy-3-methoxycinnamaldehyde (4H3MC) [47], hesperidin [48], and andrographolide [49] were found to inhibit melanogenesis in B16F10 melanoma cells, as well as in human melanocytes. However, there are several controversies, regarding the effect of quercetin (3,3,4,5,7-pentahydrosyflavone), based on in vitro and human trials. Quercetin, a popular flavonoid aglycone derived from various fruits and vegetables, is a potent melanogenesis and tyrosinase inhibitor in murine B16F10 melanoma cells [50,51,52,53]. However, some reports have indicated quercetin to have an opposite effect in human melanoma cells [54]. For these reasons, quercetin is widely debated, considering the potential application in the cosmetic field. Likewise, although further studies are required to elucidate an anti-melanogenic effect of ganodermanondiol in human melanocytes, this compound warrants further investigation for development as a potential ingredient in cosmetic applications. Furthermore, several reports suggest that the inhibition of melanogenesis might represent a valid therapeutic target for the treatment of melanotic melanomas [6,55,56,57]. Thus, ganodermanondiol may also be a potentially useful therapeutic agent for the management of melanoma.

## Abbreviations

α-MSH	alpha-melanocyte-stimulating hormone
B16F10	*Mus musculus* skin melanoma cell
HQ	hydroquinone
l-DOPA	l-3,4-dihydroxyphenylalanine
MITF	microphthalmia-associated transcription factor
MTT	3-(4,5-dimethylthiazol-2-yl)-2,5-diphenyltetrazolium bromide
TRP	tyrosinase-related protein
ERK	extracellular signal-regulated kinase
JNK	c-Jun N-terminal kinase
MAPK	mitogen-activated protein kinase
CREB	cyclic adenosine monophosphate (cAMP) response element binding protein
